# Improve the management of cancer-related cognitive impairment in clinical settings: a European Delphi study

**DOI:** 10.1007/s11764-023-01436-8

**Published:** 2023-11-07

**Authors:** Mylène Duivon, Marie Lange, Giulia Binarelli, Johan Lefel, Isabelle Hardy-Léger, Régine Kiasuwa-Mbengi, Jean-Baptiste Méric, Cécile Charles, Florence Joly

**Affiliations:** 1 Normandie Univ, UNICAEN, INSERM, ANTICIPE, 14000 Caen, France; 2 Clinical Research Department, Centre François Baclesse, 14000 Caen, France; 3 Cancer & Cognition Platform, Ligue Contre le Cancer, 14000 Caen, France; 4 Care Support Department, Centre Henri Becquerel, 76000 Rouen, France; 5 Hopital Cochin, 75014 Paris, France; 6 Department of Public Health and Epidemiology, Belgian Cancer Centre, Sciensano, Brussels, Belgium; 7 Public Health Division, National Cancer Institute, 52 Avenue André Morizet, 92100 Boulogne-Billancourt, France; 8 Bordeaux Population Health Research Center, University of Bordeaux, Bordeaux, France; 9 Medical Oncology Department, CHU de Caen, 14000 Caen, France

**Keywords:** Cancer, Cancer-related cognitive impairment, Screening, Non-pharmacological interventions, Cognitive rehabilitation, Delphi method

## Abstract

**Purpose:**

Cancer-related cognitive impairment (CRCI) is under-addressed by healthcare professionals owing to a lack of clinical management guidelines. This European Delphi study proposes recommendations to healthcare professionals for the management of CRCI in patients with non-central nervous system (non-CNS) cancers.

**Methods:**

Twenty-two recommendations were developed based on a literature review and authors’ clinical experience, split into three categories: screening, cognitive assessment, intervention. The survey included European professionals, experts in CRCI. The Delphi method was used: experts rated the clinical relevancy of recommendations on a 9-point Likert scale in three rounds. A recommendation was accepted if all votes were between 7 and 9. Recommendations not accepted in round 1 and round 2 were deleted, or modified and rated in round 3.

**Results:**

Eighteen professionals (psychologists, physicians, researchers) voted and accepted 15 recommendations. Experts recommended the systematic screening of CRCI, followed by a short objective cognitive assessment, if complaints screened. A comprehensive evaluation is recommended if CRCI persists 6 months post-treatment. Cognitive rehabilitation, physical activity, meditative-movement therapy, and multimodal intervention should be offered. Recommendations about frequency and duration of interventions, the professional to administer cognitive rehabilitation and the use of meditation and cognitive training without psychoeducation were not accepted.

**Conclusions:**

This survey provides 15 recommendations to assist healthcare professionals in detecting, assessing and offering interventions for CRCI.

**Implications for cancer survivors:**

These recommendations should be included in supportive care to help healthcare professionals to detect CRCI and propose the best available intervention for patients with cognitive complaints. Developing CRCI management in clinical settings would improve patients’ quality of life.

## Introduction

Cancer-related cognitive impairment (CRCI) is one of the main symptoms experienced by patients with non-central nervous system (CNS) cancers and affecting their quality of life [1]. It refers to cognitive difficulties experienced by cancer survivors such as difficulty remembering things and difficulty focusing on tasks [2, 3].

Subjective cognitive difficulties, aka cognitive complaints, assessed by self-reported questionnaires are usually representative of difficulties encountered in daily life. Cognitive complaints are often related with psychological factors (e.g., anxiety and depression), fatigue, pain, and sleep difficulties [4–6] but are not systematically related with objective cognitive impairment [7]. Objective impairment is detected during neuropsychological evaluations by using cognitive tests. It mainly concerns attention, memory, executive functions, and processing speed [8, 9]. Around 30% of patients experience an objective overall cognitive impairment before and after adjuvant treatment [5, 10]. The term CRCI is used in this study to characterize both subjective and objective cognitive difficulties observed in survivors with non-CNS cancer.

CRCI can last for several years for some patients [11] and thus affects the resumption of daily life activities. For example, a large web-based survey conducted among 1610 cancer survivors found that CRCI had an impact on return to work for 76% of survivors who had cognitive complaints [12]. Furthermore, 75% of cancer survivors reporting CRCI in that study would have liked to receive help such as cognitive training, psychological support and physical rehabilitation.

CRCI has been recognized for over 20 years, with recommendations for screening and cognitive assessment established by expert societies such as the International Cancer and Cognition Task Force [13–16]. Although used in research studies to harmonize findings, these recommendations are rarely followed by healthcare professionals and are not always applicable in clinical settings. In clinical practice, a neuropsychological assessment theoretically allows a patient to be offered individualized management of cognitive difficulties. Yet neuropsychological tests lack sensitivity to detect subtle cognitive difficulties affecting a patient’s daily routine and quality of life, which are better detected with self-report questionnaires. Neuropsychological tests are classically used to evaluate the efficiency of interventions, but the inclusion of patients in intervention groups relies on capturing cognitive complaints [17, 18]. Furthermore, the aim in research is to establish a standardized intervention rather than the most appropriate individualized intervention. Thus, clinical practice needs recommendations different than those used in research studies on interventions.

Over the past decade, the advent of research on interventions to improve CRCI has led to several systematic reviews and meta-analyses showing the efficacy of non-pharmacological interventions [19, 20], whereas no evidence to date supports pharmacological interventions [21]. Recent research has therefore focused mostly on non-pharmacological interventions and, until now, cognitive training, cognitive rehabilitation and physical activities seem the most effective [22–26]. Although some local schemes offering non-pharmacological interventions for CRCI have been reported in European countries, they are scarce in clinical settings because of the lack of financial resources and trained health professionals [27, 28].

To summarize, there is a lack of clinical recommendations and initiatives in Europe to detect and offer adapted interventions to patients with cognitive complaints, so health professionals and patients are at loss when they arise [2, 29, 30]. A recent survey aiming to raise awareness among European public authorities about CRCI in patients with non-CNS cancers showed that CRCI is still insufficiently understood and considered by public authorities and healthcare professionals [28]. Although CRCI is starting to be included in survivorship programs [31–33], numerous clinicians (e.g., oncologists, nurses, general practitioners) are not aware of the existence of these cognitive difficulties [30]. As a result, patients lack information about CRCI, which goes undetected and unmanaged. In the Europe’s Beating Cancer plan [34], the current main framework for cancer control at the European Union level, very little attention is given to survivorship rehabilitation programs and their content, such as CRCI.

Management of CRCI (screening, evaluation, intervention) is therefore a major challenge in clinical practice, and healthcare professionals require concrete answers and applications to be able to support patients, especially once their treatment has been completed. In 2021, a collaboration with the innovative Partnership for Action Against Cancer (iPAAC) Joint Action, a scheme was undertaken to create guidelines to help public authorities to deal with CRCI [28]. Discussions with several stakeholders from public authorities, associations, and medical and research structures in Europe highlighted the need to guide health professionals. As a result of this collaboration, a consultation on the subject was launched with European experts. Recommendations were drafted on the basis of a literature review and knowledge collected in clinical settings during the iPAAC project. Then, the Delphi method was used to establish a clinical consensus of experts on the issue of cancer and cognition for the management of CRCI.

## Methods

The study used the Delphi methodology based on the RAND method [35]. This is a series of iterative questionnaires determining the clinical relevancy of recommendations to be applied in clinical settings. The aim of this study was to propose expert-validated recommendations for health professionals to help them manage CRCI.

### Initiation of the Delphi study

The authors of the recommendations (MD, GB, ML, JLF, IHL, CC, and FJ) were psychologists (*n*=5), oncologists (*n*=1), and/or researchers (*n*=4), all studying CRCI. Each participated in a project under the iPAAC Joint Action to propose recommendations for public authorities to help them organize the management of CRCI. The project included a field survey (interviews with public authorities, associations, expert societies) and a literature review of interventions for CRCI. Results were summarized and used to establish recommendations for public authorities. These were then debated during a workshop including authors, the stakeholders interviewed during the field survey and the public authorities. The project highlighted the need to establish recommendations that clinicians could implement in clinical settings [28]. The recommendations therefore focused on the entire care itinerary, from screening up to and including the intervention.

### Establishment of recommendations

The recommendations were based on the data collected (scientific literature review, non-scientific review, interviews with health professionals) on CRCI management during summer 2021 by MD for the iPAAC project [28] and on the clinical experience of the authors (GB, ML, JLF, IHL, CC, FJ). The scientific literature search was made on the Web of Science, Pubmed (MEDLINE), University of Montreal, Wiley, and ScienceDirect databases (for the narrative review see [27]). The primary focus (articles used to develop the recommendations) was interventions to manage CRCI in adults with non-CNS cancers (e.g., cognitive training, rehabilitation, physical activity, meditation, yoga, cognitive behavioral therapy). For details on data collection, see iPAAC guide [28]. The non-scientific review with a search engine (Google) and interviews with stakeholders during the iPAAC project covered patient management more broadly in five topics [28]:

- informing about CRCI;
- screening CRCI and evaluating cognitive functioning;
- organizing CRCI management (orienting, coordinating, offering interventions);
- managing CRCI to allow the return to work;
- identifying health professionals to be involved in CRCI management and training them.

Recommendations about screening and the evaluation of cognitive difficulties were based on the scientific literature review, complemented by the authors’ personal digital libraries on CRCI and data of health professionals’ iPAAC interviews.

Recommendations on interventions were based on the literature review (updated in 2022) and health professionals’ interviews in the iPAAC survey. Types of interventions were selected according to scientific evidence-based results, i.e., most studied interventions with evidence about their efficacy in improving cognitive complaints and/or cognitive functioning. They were defined as follows:

- Cognitive training: improving cognitive difficulties using repetitive and sustained exercises (frequently computerized) with incremental difficulty based on the patient’s performance.
- Psycho-education: education/information about cognitive functioning, how it might be affected by cancer and its treatments and how to deal with it. Psycho-education gives some advice and strategies to reduce cognitive difficulties encountered in daily life and thus improve quality of life.
- Cognitive rehabilitation: interventions combining psycho-education, cognitive training, and/or cognitive behavioral therapy, in order to improve cognitive difficulties observed in daily life. Cognitive behavioral therapy proposes new behaviors to adopt, to improve or compensate for a specific function.
- Physical activity: physical exercises such as aerobic exercises and walking.
- Mindfulness / Meditation: breathing exercises, visualization and other techniques to develop attention, awareness, relaxation.
- Meditative movement therapy: gentle exercises combined with relaxation such as yoga.
- Multimodal interventions: combination of several approaches among those previously mentioned.

### Participants

Experts were identified through the European iPAAC project, cancer associations (e.g*.*, *Ligue Contre le Cancer*), studies conducted on CRCI management in Europe, and the authors’ network of professional contacts. Participants were either healthcare professionals (e.g., clinical psychologists) involved in cancer survivorship care, physicians (e.g., oncologists) seeing cancer patients and aware of the issue of cognitive disorders, or researchers working in the field of cancer and cognition. Their status could be multiple, i.e., both healthcare professional and/or physician and/or researcher. They received an e-mail explaining the aim and procedure of the Delphi method to be able to participate in the survey. Participants who agreed to participate received the link to complete each survey online.

Characteristics of participants were reported: professional activity, country of professional activity, number of years involved in this field, level of involvement in survivorship care, association or other membership, and involvement in the drafting of guidelines dedicated to cancer and cognition.

### Survey development

After validation by the ethics committee of the University Hospital of Caen, the software LimeSurvey, provided by the University of Caen Normandy, was used to create the questionnaires. Each recommendation or group of recommendations was preceded by a short state of the art and a short definition of the intervention (see the section ‘Establishment of recommendations’). A 9-point Likert scale was used to rate the clinical relevance and feasibility, including “1-Not agree at all”, “5-Undecided”, and “9-Totally agree”. Participants had to specify to what extent they thought the recommendation was clinically relevant (i.e., useful and necessary for patients) and to what extent the recommendation could be feasible in their institution or another establishment (to be specified in the comment box). If the participant rated the recommendation 6 or less, a mandatory text box appeared to justify his/her disagreement. An optional text box was also available so that participants agreeing with the recommendation could add further information concerning their clinical experience, references, and discuss the suggestions associated with the recommendation. A tick box “I am not able to respond” was also available for participants who considered that the recommendation was not in their area of expertise.

### Analysis

Characteristics of participants, i.e., country of professional activity, professional status, and previous participation in iPAAC, were compared with Chi² tests between individuals who did or did not participate in the Delphi survey.

A table of descriptive statistics was created for each round including: the number of participants, the median and range of scores, the number and percentage of participants agreeing and disagreeing. The percentages of responses were categorized as follows: “agree” percentage of participants who voted between 7 and 9; “neutral” who voted between 4 and 6, “disagree” who voted between 1 and 3; and “not able to respond.” The proposal judgement based on RAND recommendations [35, 36] was rated according to the median (determines whether the recommendation is appropriate, inappropriate, or uncertain) and the voting range (determines if it is strong or relative) as follows:

- Appropriate with strong agreement: median ≥ 7 [7–9]
- Appropriate with relative agreement: median ≥ 7 [5–9]
- Inappropriate with strong agreement: median ≤ 3 [1–3]
- Inappropriate with relative agreement: median ≤ 3.5 [1–5]
- Uncertain with indecisiveness: 4 ≤ median ≤ 6.5 [1–9]
- Uncertain with a lack of consensus: other cases

The recommendation was accepted only if a consensus was reached, i.e., only when the first condition (“Appropriate with strong agreement”) was met.

#### Round 1 - Survey

The first round required participants to rate the clinical relevancy and the clinical feasibility of the recommendations, each on a 9-point Likert scale. Suggestions of applications in clinical settings, i.e., concrete examples, were associated with some recommendations to help participants to understand its applicability. Recommendations were accepted if all participants voted 7 or more. No consensus was expected for clinical feasibility, which depends on several factors from one country and clinical establishment to another. However, the suggestions of participants (mandatory and optional text boxes) concerning clinical relevancy and clinical feasibility were essential to discuss the recommendations and provide the appropriate suggestions in the discussion of this study.

#### Round 2 - Survey

The second round concerned the recommendations for which no consensus was obtained during the first round. Only clinical relevancy was evaluated in this round, with the method previously described.

To assist participants in voting, a summary of the first-round results including descriptive statistics and an anonymized summary of comments was presented before each recommendation. The summary allowed participants to become aware of the group opinion and thus to open up the discussion concerning the acceptance or not of an intention. Furthermore, each participant received a personalized e-mail with his/her previous vote.

Acceptance of recommendation was less strict in the second round as outliers could be excluded. According to the HAS (French High Health Authority) guidelines, when there are 15 to 30 participants, two outliers may be excluded if there are no missing values and 1 outlier may be excluded if 1 value is missing [36, 37]. In other cases, extreme values were not excluded. In other words, the extreme values (2 maximum) could be excluded to obtain a consensus.

#### Round 3 - Survey

In the third and last round, the recommendations for which no consensus was obtained in the second round were either deleted or modified on the basis of the participants’ comments. After the authors (MD, GB, ML, JLF, IHL, CC, FJ) had validated the new (modified) recommendations, they went to a final round of voting. As in the second round, only clinical relevancy was evaluated.

To help participants in voting, each one received a personalized e-mail with their previous vote and the summary (as previously described) of the second round to justify the deletions and modifications of the remaining recommendations.

## Results

### Participants

Thirty-nine experts were contacted and 18 accepted to participate (at least one round responded entirely) in the survey (participation rate 46%). No difference was observed concerning the category and country of residence between experts who accepted to participate and those who did not answer the invitation. Nevertheless, experts who participated in the iPAAC survey were significantly more likely to participate in this Delphi study than experts whom did not participate in the iPAAC survey (*p*<0.001). The characteristics of the 18 participants are described in Table 1. More than 60% of participants had clinical experience and daily contact with patients, including expertise on the organization of clinical facilities.

**Table 1:** Characteristics of the 18 participants

Characteristics	*n* (%)
Category	
Physician^a^	5 (28)
Psychologist^b^	3 (17)
Researcher^c^	4 (22)
Physician + researcher	2 (11)
Psychologist + researcher	4 (22)
Years of experiences in « Cancer and Cognition » domain	
≤5 years	4 (22)
6 < years ≤ 10	7 (39)
> 10 years	7 (39)
Involvement in patient management	
Clinical relationship with patients	11 (61)
Research involving a relationship with patients	3 (17)
Academic research	4 (22)
Member of learned societies (e.g., ICCTF, AFSOS, SNLF)	14 (78)
Involved in creating previous guidelines concerning « Cancer and Cognition » (AFSOS and ICCTF)	2 (11)
➔ iPAAC	13 (72)
Country of residence	
France	12 (67)
Belgium	2 (11)
Denmark	2 (11)
Portugal	1 (6)
Netherlands	1 (6)

^a^Oncologist, geriatrician, hematologist, psychiatrist, physical and rehabilitation medicine doctor

^b^Clinical psychologist, work psychologist, and neuropsychologist

^c^Post-doc researcher, associate professor and professor.

*AFSOS* French association of oncological support, *ICCTF* International Cognition and Cancer Task Force, *SNLF* Neuropsychological Society of French Language, *IPAAC* International Partnership for Action Against Cancer

### Delphi survey

Table 2 summarizes the recommendations arising from the three rounds.

**Table 2:** Summary of results of three rounds of voting

Recommendations		Round 1			Round 2			Round 3		
		*n*	median [range] % agree	Rating	*n*	median [range] % agree	Rating^a^ (final *n*)	*n*	median [range] % agree	Rating^a^ (final *n*)
**Screening of cognitive complaints**										
✓ **Recommendation 1**	Every patient should be screened for cognitive complaints *Suggestions:* *- At the time of diagnosis* *- During adjuvant treatments* *- At the end of adjuvant treatments*	18	9 [7–9] 100%	**Appropriate, strong agreement**	NA			NA		
✓ **Recommendation 2**	Screening of cognitive complaints should be realized with simple questions from validated questionnaires *Suggestions:* *- Have you had concerns about memory or concentration in the past week, including today? (NCCN distress thermometer)* *- Have you had difficulty in concentrating on things, like reading a newspaper or watching television? (EORTC-QLQ-C30)* *- Do you find your thinking has been slow? (FACT-Cog V3)*	18	7.5 [3–9] 61%	Uncertain, lack of consensus	18	8 [5–9] 94%	**Appropriate, strong agreement (17)**	NA		
✓ **Recommendation 3**	If cognitive complaints have been screened, their severity and their impact on daily living should be assessed with a validated self-report questionnaire *Suggestions:* *- PROMIS Cognitive function 8 items* *- FACT-Cog*	18	8 [6–9] 94%	Appropriate, relative agreement	18	8.5 [5–9] 89%	**Appropriate, strong agreement (16)**	NA		
**Cognitive evaluation**										
✓ **Recommendation 4**	In case of cognitive complaints, a short objective cognitive evaluation should be performed *Suggestions:* *- Orientation toward a pivot nurse (or other corresponding clinician according to the country and center)* *- Evaluation with a cognitive screening test such as the Montreal Cognitive Assessment (MOCA)* *- Assessment with some cognitive tests of the main impaired cognitive domains in cancer patients (memory, executive functions, attention, processing speed)*	18	8 [1–9] 83%	Uncertain, lack of consensus	18	8 [6–9] 94%	**Appropriate, strong agreement (17)**	NA		
↓ **Recommendation 5**	In the event of cognitive impairments detected with a short objective cognitive evaluation (Recommendation 4), patients should be addressed to a psychologist for a comprehensive cognitive evaluation. This assessment will identify oncological or neurological causes of cognitive impairment that would require further medical testing and appropriate management. *Suggestions:* *- Referral to a psychologist specialized in neuropsychology* *- The psychologist can rely on validated neuropsychological test batteries (for a review, see Lange et al., 2019)*	18	9 [1–9] 78%	Uncertain, lack of consensus	18	8 [1–9] 83%	Uncertain, lack of consensus	Modified		
✓ **New recommendation 5**	➔ If persistence of CRCI detected around 6 months post-treatment (such as chemotherapy), the patient should undergo a medical consultation to explore etiology of cognitive impairment. This consultation could be complemented by a neuropsychological assessment with a neuropsychologist.	NA			NA			17	8 [4–9] 88%	**Appropriate, strong agreement (15)**
✓ **Recommendation 6**	To adapt the support to patients’ needs, factors associated with cognitive impairments should be assessed, i.e., mainly anxiety, depression, fatigue, sleep, and pain	17	9 [7–9] 100%	**Appropriate, strong agreement**	NA			NA		
✓ **Recommendation 7**	In the event of significant cognitive complaints and/or objective cognitive impairments related to cancer and/or its treatments, non-pharmacological supervised support should be offered to patients	16	9 [7–9] 94%	**Appropriate, strong agreement**	NA			NA		
**Intervention: cognitive training alone**										
*x* **Recommendation 8**	Cognitive training should be offered to all cancer patients with CRCI, to reduce cognitive complaints and/or improve cognitive performances	17	7 [3–9] 65%	Uncertain, lack of consensus	18	7 [4–9] 83%	Uncertain, lack of consensus	**Deleted**		
*x* **Recommendation 9**	Duration of the cognitive training program ⇒ at least 2 months *Suggestions:* *- 4 months* *- 6 months*	16	7 [1–9] 65%	Uncertain, lack of consensus	18	7 [3–9] 72%	Uncertain, lack of consensus	**Deleted**		
*x* **Recommendation 10**	Frequency of the cognitive training sessions ⇒ at least 2 per week *Suggestions:* *- 3 per week* *- 4 per week*	16	7 [1–9] 88%	Uncertain, lack of consensus	17	7 [4–9] 67%	Uncertain, lack of consensus	**Deleted**		
*x* **Recommendation 11**	Duration of the cognitive training sessions ⇒ at least 30 min *Suggestions:* *- 45min* *- 1h*	16	7 [1–9] 82%	Uncertain, lack of consensus	17	7 [1–9] 78%	Uncertain, lack of consensus	**Deleted**		
*x* **Recommendation 12**	Cognitive training should be supervised by a psychologist or another clinician, trained in neuropsychology, and with knowledge about the intervention used *Suggestions:* *- On site: supervised by a psychologist* *- Home-based: supervised through phone calls*	17	8 [1–9] 82%	Uncertain, lack of consensus	18	8.5 [1–9] 89%	Uncertain, lack of consensus	**Deleted**		
**Intervention: psycho-education**										
↓ **Recommendation 13**	At least two psycho-education sessions should be offered to all cancer patients with cognitive complaints *Suggestions:* *- 3 sessions* *- 4 sessions*	16	8 [4–9] 71%	Uncertain, lack of consensus	16	8 [4–9] 78%	Uncertain, lack of consensus	Modified		
✓ **New recommendation 13**	➔ At least one psycho-education session should be offered to all cancer patients with cognitive complaints	NA			NA			17	8 [7–9] 100%	**Appropriate, strong agreement (17)**
**Intervention: cognitive rehabilitation (psycho-education + cognitive training)**										
✓ **Recommendation 14**	A cognitive rehabilitation program, i.e., combining psycho-education with cognitive training and/or cognitive behavioral therapy (for CRCI), should be offered to all cancer patients with CRCI, to improve complaints and cognitive functioning in daily life activities	15	8 [7–9] 88%	**Appropriate, strong agreement**	NA			NA		
↓ **Recommendation 15**	Duration of the cognitive rehabilitation program ⇒ at least 4 weeks *Suggestions:* *- 5 weeks* *- 6 weeks*	15	8 [1–9] 82%	Uncertain, lack of consensus	16	8 [1–9] 78%	Uncertain, lack of consensus	Modified		
✓ **New recommendation 15**	➔ Frequency and duration of the sessions, and duration of the program should be adapted to the patient and the establishment where intervention takes place	NA			NA			17	8 [5–9] 94%	**Appropriate, strong agreement (16)**
↓ **Recommendation 16**	Frequency of the sessions of cognitive rehabilitation ⇒ at least 1 per week (depending on the program) *Suggestions:* *- One session per week combining psycho-education then cognitive training or cognitive behavioral therapy* *- Two separate sessions per week (e.g., one psycho-education session and one cognitive training session)* *- One session per week on-site and homework (e.g., psycho-education on-site and home-based cognitive training)*	15	7 [3–9] 76%	Uncertain, lack of consensus	16	8 [3–9] 83%	Uncertain, lack of consensus	Modified		
✓ **New recommendation 16**	➔ On-site sessions and/or home-based exercises should be offered	NA			NA			17	8 [3–9] 88%	**Appropriate, strong agreement (15)**
↓ **Recommendation 17**	Duration of cognitive rehabilitation sessions ⇒ at least 30 min (depending on the number/distribution of sessions) *Suggestions:* *- Several sessions of 30min (one approach by session)* *- One session of 2 h (combining approaches)*	14	7 [2–9] 71%	Uncertain, lack of consensus	17	8 [1–9] 83%	Uncertain, lack of consensus	Modified		
✓ **New recommendation 17**	➔ Duration of cognitive rehabilitation sessions (cognitive training + psycho-education) ⇒ at least 45 min	NA			NA			16	8 [7–9] 100%	**Appropriate, strong agreement (16)**
↓ **Recommendation 18**	Psycho-education and cognitive behavioral therapy have to be supervised by a psychologist	17	9 [3–9] 88%	Uncertain, lack of consensus	17	9 [1–9] 78%	Uncertain, lack of consensus	Modified		
✓ **New recommendation 18**	➔ Cognitive rehabilitation (psycho-education ± cognitive training) should be administered by a therapist trained in cognitive rehabilitation (e.g., occupational or speech therapist, nurse, medical doctor) preferably with training in psychology (e.g., neuropsychologist)	NA			NA			17	9 [1–9] 76%	**Uncertain lack of consensus** **(NA)**
**Intervention: physical activity**										
✓ **Recommendation 19**	Whatever cognitive intervention already offered, physical activity should be offered to all cancer patients with CRCI, to improve cognitive performances and health status *Suggestions:* *- Propose physical activity program already available in cancer survivorship program* *- Refer patient to associations or other establishments offering physical activity sessions adapted to cancer survivors*	17	9 [7–9] 100%	**Appropriate, strong agreement**	NA			NA		
**Intervention: mindfulness/meditation**										
↓ **Recommendation 20**	Meditation/mindfulness may be considered as a therapeutic option and offered to patients with CRCI, according to their preferences, to improve their cognitive functioning and psychological status	16	7.5 [6–9] 88%	Appropriate, relative agreement	17	7 [2–9] 78%	Uncertain, lack of consensus	Modified		
*x* **New recommendation 20**	➔ Mindfulness/Meditation may be considered as a therapeutic option and offered to patients with CRCI, in combination with cognitive rehabilitation and according to their preferences, to improve their cognitive functioning and psychological status							17	7 [3–9] 82%	**Uncertain lack of consensus** **(NA)**
**Intervention: meditative movement therapies**										
✓ **Recommendation 21**	Meditative movement therapies may be considered as a therapeutic option and offered to patients with CRCI, according to their preferences, to improve cognitive functioning and quality of life	16	7 [7–9] 94%	**Appropriate, strong agreement**	NA			NA		
**Intervention: multimodal interventions**										
✓ **Recommendation 22**	Multimodal interventions, i.e., cognitive intervention combined with other approaches chosen according to patients’ preferences and needs (among physical activity and mind body therapies), are preferable to improve CRCI and associated symptoms	17	9 [5–9] 94%	Appropriate, relative agreement	18	8.5 [5–9] 94%	**Appropriate, strong agreement (17)**	NA		

% agree: percentage of participants who agree with the recommendation, i.e., vote ≥7 on the whole sample; Rating: judgement of the recommendation according to median and range; ^a^: Rating judgment after excluding extreme values (according to RAND guidelines); final *n*= number of participants after excluding extreme values. In bold, rating of final decision

✓ Recommendation accepted

*x* Recommendation deleted

↓ Recommendation modified

#### Round 1

Among the 22 recommendations, six reached a consensus (judged as appropriate with strong agreement) and were accepted (see Table 2): Recommendations 1, 6, 7, 14, 19, 21.

For the remaining recommendations, three were judged appropriate with relative agreement, and 13 were judged uncertain with a lack of consensus (see Table 2). Therefore, these 16 recommendations went to a second round of voting.

#### Round 2

In the second round, four recommendations were judged as appropriate with relative agreement. After excluding extreme values, the recommendations 2, 3, 4, and 22 were accepted (see Table 2).

The remaining 12 recommendations were judged as uncertain, with a lack of consensus. Following the experts’ comments, the recommendations for which no consensus was reached were either removed or modified to be voted in the third round. The five recommendations related to cognitive training “alone” were removed, i.e., recommendations 8 to 12 (see Table 2).

Concerning the seven remaining recommendations, they were modified according to the experts’ suggestions (see Table 2).

#### Round 3

In the final round, seven recommendations had to be voted again to reach a consensus. Two recommendations were judged as appropriate with strong agreement, one recommendation as appropriate with relative agreement, and the four others as uncertain with a lack of consensus. After excluding the extreme values, three more recommendations reached consensus. Thus, the recommendations 5, 13, 15, 16, and 17were accepted (see Table 2).

As this was the final round, the recommendations 18 and 20 that did not reach consensus were deleted.

The final recommendations that reached consensus during the three rounds are listed in Table 3.

**Table 3:** Final list of accepted recommendations

Recommandations	
Screening of cognitive complaints	
Recommendation 1	Every patient should be screened for cognitive complaints
Recommendation 2	Screening of cognitive complaints should be realized with simple questions from validated questionnaires
Recommendation 3	If cognitive complaints have been screened, their severity and their impact on daily living should be assessed with a validated self-report questionnaire
Cognitive assessment	
Recommendation 4	In the event of cognitive complaints, a short objective cognitive evaluation should be performed
Recommendation 5	If persistence of CRCI detected around 6 months post-treatment (such as chemotherapy), the patient should undergo a medical consultation to explore etiology of cognitive impairment. This consultation could be complemented by a neuropsychological assessment with a neuropsychologist.
Recommendation 6	To adapt the support to patients’ needs, factors associated with cognitive impairment should be assessed, i.e., mainly anxiety, depression, fatigue, sleep, and pain
Recommendation 7	In the event of significant cognitive complaints and/or objective cognitive impairment related to cancer and/or its treatments, non-pharmacological supervised supports should be offered to patients
Psycho-education	
Recommendation 13	At least one psycho-education session should be offered to all cancer patients with cognitive complaints
Cognitive rehabilitation	
Recommendation 14	A cognitive rehabilitation program, i.e., combining psycho-education with cognitive training and/or cognitive behavioral therapy (for CRCI), should be offered to all cancer patients with CRCI, to improve complaints and cognitive functioning in daily life activities
Recommendation 15	Frequency and duration of the sessions, and duration of the program should be adapted to the patient and the establishment where the intervention takes place
Recommendation 16	On-site sessions and/or home-based exercises should be offered
Recommendation 17	Duration of the session of cognitive rehabilitation (cognitive training + psycho-education) should be at least 45 min
Physical activity	
Recommendation 19	Whatever cognitive intervention already offered, physical activity should be offered to all cancer patients with CRCI, to improve cognitive performances and health status
Meditative movement therapies	
Recommendation 21	Meditative movement therapies (i.e., gentle exercises combined with relaxation such as yoga) may be considered as a therapeutic option and offered to patients with CRCI, according to their preferences, to improve cognitive functioning and quality of life
Multimodal interventions	
Recommendation 22	Multimodal interventions, i.e., cognitive intervention combined with other approaches chosen according to patients’ preferences and needs (among physical activity and mind body therapies), are preferable to improve CRCI and associated symptoms

## Discussion

In Europe, management of CRCI in patients with non-CNS cancers is a growing concern. Therefore, this Delphi study aimed to develop guidelines for supporting patients with CRCI based on scientific evidence and expert knowledge. Eighteen experts in CRCI (psychologists, physicians and researchers) approved 15 out of 22 recommendations.

In summary, experts recommended systematically screening for CRCI followed by a short objective cognitive assessment if cognitive complaints screened. A comprehensive evaluation (including a neuropsychological assessment and an evaluation of co-factors) should be performed if CRCI persists for more than 6 months post-treatment. Non-pharmacological supervised interventions should be offered, prioritizing cognitive rehabilitation (including psycho-education), physical activity, meditative movement therapies, or multimodal intervention.

### Screening for CRCI

Patients should be systematically screened for cognitive impairment (Recommendation 1) with simple questions from validated questionnaires (Recommendation 2). In the literature, the large majority of studies on interventions included patients with perceived cognitive difficulties assessed trough self-report questionnaires (e.g., FACT-Cog, EORTC QLQ C30, CFQ) [22, 23] or using simple questions (e.g., “do you have concerns about your memory or other thinking abilities following cancer treatment?” [18]). In clinical setting for example, the Capital Region of Denmark’s working group [38] and Regional Cancer Centers in Sweden [39] recommend using questionnaires with checkboxes such as the NCCN distress thermometer (listing a variety of symptoms including concentration/memory) to detect the need for rehabilitative care. Then if cognitive complaints are detected with simple questions (see suggestions Table 2), their severity and their impact on daily living should be assessed with a validated self-report questionnaire (Recommendation 3). The choice of the self-report questionnaire depends on the time available and the language involved. The panel of experts mainly proposed the FACT-Cog [15] which has several advantages: available in several languages; evaluates the impact of cognitive difficulties on quality of life; specific to patients with cancer as it has been developed and validated by cancer patients. Another questionnaire validated in cancer patients and recommended by the Cancer Neuroscience Initiative Working group [16] is the Patient-Reported Outcome Measurement Information System Cognitive Function, short form [40, 41].

Ideally, screening should be performed throughout the healthcare pathway of the patient, i.e., at the time of diagnosis, during adjuvant treatments, and at the end of adjuvant treatments (suggestions of Recommendation 1, see Table 2). If there are some initiatives in clinical settings to screen symptoms and supportive care needs, it is not yet generalized and cognitive difficulties have not been well identified. Thus, regarding the resources available for now in clinical settings, the experts emphasized the need for evaluation in the months following the end of adjuvant treatments (radiotherapy and/or chemotherapy). The progressive introduction of digital tools in the follow-up of patients, such as app to assess cancer treatments side-effects, will be a lever to screen and follow more easily cognitive complaints.

### Cognitive assessment

In the event of cognitive complaints attested by a self-report questionnaire (screening), it is recommended to perform a short objective cognitive evaluation (Recommendation 4) before the comprehensive neuropsychological evaluation. The choice of test will depend on several factors such as the age of the patient, the health professional available (e.g., trained nurse or neuropsychologist) and the time needed to perform the evaluation. According to the literature and our experience of clinical settings, the following tests would be appropriate: the MOCA [42] or the combination of The Hopkins Verbal Learning Test-Revised [43], the Trail-Making Test [44], and the Controlled Oral Word Association [45] recommended by the ICCTF [14]. Although cognitive screening tests (MOCA and MMSE) lack of sensitivity to detect subtle cognitive difficulties encountered by patients, the MOCA seems more sensitive than MMSE in older cancer patients [46]. The MOCA can be administered by every trained and attested professional, whereas the other tests require a psychologist specialized in neuropsychology. New screening tools (inspired by the MOCA with a lower ceiling effect) have also been created in an attempt to provide a tool fast to use in clinical settings and sensitive to CRCI [47].

Then, according to the experts, if persistent CRCI is detected six months after treatments, whether or not an intervention has been performed, a medical check-up should be done. Thus, the etiology of cognitive difficulties such as the use of psychotropic medications, biological disorders (e.g., anemia), or comorbidities, should be evaluated and a neuropsychological evaluation performed, if needed (new recommendation 5). The choice of cognitive tests should be made by the neuropsychologist who, if needed, can rely on the scientific literature about CRCI [13]. This consultation should include the evaluation of associated factors such as anxiety, depression, sleep disorders and fatigue (Recommendation 6). The arbitrary date of six months has been proposed based on the literature and expert comments, considering that CRCI is supposed to decrease six months after treatments [48, 49]. Obviously, it is not required to wait 6 months if an evaluation seems necessary, but it is recommended to avoid performing an evaluation in the weeks following the end of treatment because of the persistence of symptoms that are supposed to decrease progressively.

CRCI is characterized by cognitive complaints not systematically related with neuropsychological assessment [7]. Although neuropsychological evaluation is essential to the differential diagnosis and to determine the domains impaired, it should not be a prerequisite for having access to an intervention.

### Interventions

As soon as a patient has cognitive complaints and irrespective of their confirmation by objective cognitive tests, a non-pharmacological supervised intervention should be offered (Recommendation 7). In the scientific literature on non-pharmacological interventions, computerized cognitive training seems to be widely studied and beneficial for improving CRCI [23, 50], especially improving verbal memory and processing speed [51]. However, no consensus has been found on its use without the association with psycho-education. According to the comments of the first and second rounds, the main reason of disagreement was that cognitive training alone is not effective in improving the difficulties encountered on a daily basis and must be accompanied by psycho-education. Moreover, the lack of standardization of training and the heterogeneity observed in clinical settings makes it difficult to reach a consensus on what cognitive training should comprise (e.g., duration and frequency of the sessions). Thus, the five recommendations related to cognitive training “alone” were removed. On the other hand, the experts recommended offering at least one session of psycho-education for patients with cognitive complaints [52] (Recommendation 13). As defined in the method section, this session is essential to reassuring patients by informing about “normal” cognitive functioning and CRCI but also offering some advices to reduce difficulties encountered in daily life.

Cognitive rehabilitation, i.e., programs combining psycho-education with cognitive training [24] or with cognitive-behavioral therapy [53, 54], was recommended by the experts (Recommendation 14). According to the network meta-analysis by Cheng et al. (2022), each approach focusing on cognition (cognitive training, psychoeducation, and cognitive rehabilitation) is beneficial for particular cognitive domains. Combining these approaches would therefore improve cognition in both subjective and objective ways [51]. However, owing to the heterogeneity of studies, the choice of the frequency and duration of interventions is down to the healthcare professional’s preference (Recommendation 15). On-site sessions combined with home-based exercises should be offered in first instance to promote follow-up (Recommendation 16) with a minimum duration of 45min of combined psycho-education and cognitive training (Recommendation 17). No consensus was reached concerning the professional who should perform the cognitive rehabilitation (Recommendation 18, deleted). Indeed, depending on the health organizations and the countries, different professionals might possess the skills needed to administer cognitive rehabilitation. They should be chosen according to their specialty and the impairments observed. Psychologists specialized in neuropsychology and/or cognitive rehabilitation should be prioritized to administer cognitive rehabilitation. Speech therapists specialized in neuropsychology may also administer cognitive rehabilitation but should rather be recommended in the event of language impairments such as occupational therapists for motor impairments. The experts also suggested that psycho-education could be administered by oncologists, nurses, medical doctors, and therapists with a specialization (university diploma) in rehabilitation or neuropsychology. This is not an exclusive list and the healthcare professionals required could differ from one country to another.

Physical activity (Recommendation 19) and “meditative movement therapies” (Recommendation 21) were recommended by the experts. Both interventions have been proven to reduce the cognitive complaints of patients with non-CNS cancers [55–58], however, knowledge of the benefits of activity on objective cognition is still limited [59]. Exercises are already offered by some associations (e.g., *Ligue Contre le Cancer* in France) and cancer centers (adapted physical activity), and several public authorities or health organizations across Europe (e.g., France, Denmark, Norway, Sweden), including the World Health Organization, encourage cancer survivors to undertake physical activity [28, 60]. Thus, physical activity in general should be promoted and recommended/prescribed by physicians in the event of cognitive complaints.

Mindfulness/meditation therapy did not reach a consensus, owing to a lack of evidence concerning its efficacy (Recommendation 20, deleted). While few studies have demonstrated an improvement in objective cognitive functioning [61, 62] it would appear to be particularly beneficial for attention [51]. Moreover, meditation/mindfulness improves cognitive complaints and psychological outcomes [63–65]. Thus, although not recommended for managing CRCI in the present survey, it might be proposed by patients’ associations and cancer centers as an additional activity to be performed at home alone or in groups to improve patients’ well-being.

Finally, multimodal interventions are recommended (Recommendation 22). Although there have only been a few studies in non-CNS cancer patients [18, 66], a recent network meta-analysis found that each type of intervention specifically improves some cognitive domains [51]. For example, cognitive training is more effective to improve verbal memory and processing speed, whereas psycho-education is more effective on executive functioning, and meditation/mindfulness on attention. Thus, combining several approaches could be more efficient to improve global cognitive functioning. Moreover, a multimodal intervention could be more effective by acting on several factors that are putatively implicated in CRCI [67].

Other therapies targeting associated factors might reduce cognitive complaints such as acupuncture for pain [68, 69] and cognitive behavioral therapy for sleep [68] or fatigue [70]. These interventions were not proposed in the recommendations owing to a lack of evidence concerning their efficiency on CRCI.

### Integration in clinical settings

Management of CRCI remains underdeveloped in clinical settings. Information to the patient and the professional is insufficient. Some initiatives are emerging at the local level thanks to patients’ associations, hospitals and cancer centers trying to respond to patients’ needs [2, 30]. However, there are several limitations: (a) no systematic offer to patients with cognitive complaints; (b) poor communication concerning management availability; (c) some interventions not free of charge; (d) when in hospital and cancer center: lack of desire of patients to return to their place of treatment; (e) few interventions for CRCI available in clinical settings; (f) limited in capacity and often prioritized for patients returning to work; (g) when on-site, especially available in large cities. These limitations reflect the social inequalities encountered in accessing CRCI management. In this study, the experts recommended physical activity and psycho-education which can be made readily available to patients (online access options [71, 72], reimbursement) and should be offered to patients as a priority if no cognitive training is available. Multimodal intervention was strongly recommended by experts during the iPAAC workshop [28], however, multimodal intervention is more difficult to implement in clinical settings owing to the requirements for coordination between different healthcare professionals and the need for greater patient participation.

### Limitations and strengths

This Delphi study has some limitations including the low number of participants, without representation of all the European countries. Our knowledge of this subject across Europe is therefore non exhaustive. As observed during the iPAAC project [28], there are few professionals who are experts in CRCI across Europe. The participation rate below 50% highlights the need to inform the health professionals involved in the care of cancer survivors. A choice was made to include only professionals who are expert in the domain of CRCI and its management, excluding *de facto* patients, and also some health professionals (e.g., nurses and general practitioners), whereas it would have been useful to have feedback from those primarily concerned or involved to better refine the recommendations. Furthermore, some points were not addressed in the vote owing to the lack of scientific evidence, such as “should the intervention be performed individually or in a group.” Although this prevents the creation of a “ready-to-use” management, we felt that it would be more appropriate for some interventions’ characteristics to be decided according to the patient and the care facility.

To our knowledge, this is the first study conducted according to the Delphi consensus method developed at RAND for CRCI management [73]. Strengths of this study also included the development of recommendations based on scientific and clinical knowledge. Recommendations were not developed at national level but based on European survey (iPAAC) and involved health professionals dealing with patients with CRCI and researchers’ experts of CRCI of several countries.

## Conclusion

These recommendations are designed for healthcare professionals to guide them in the management of CRCI, from detection, through evaluation to intervention. The existence of evidence for interventions to manage CRCI should guide decision-makers in providing resources for this type of supportive care.

This Delphi survey recommends systematically screening for CRCI. In the event of cognitive complaints, a short objective cognitive evaluation should be performed. If CRCI persists longer than six months post-treatment, experts recommend evaluating the etiology of cognitive difficulties (medical consultation and neuropsychological assessment, if needed). If CRCI is screened, non-pharmacological interventions should be offered, prioritizing cognitive rehabilitation, physical activity, meditative movement therapies or multimodal intervention. Healthcare professionals should refer patients with cognitive complaints according to local resources and the preferences and needs of patients. It is essential for the development of CRCI management that healthcare professionals become actors in strengthening and expanding existing initiatives.

Future perspectives would be for these recommendations to be relayed by public health authorities to inform all healthcare professionals dealing with non-CNS cancer patients. Interventions based on these recommendations should then be developed according to the resources available in each country.

## Data Availability

Data generated during this study are available from the corresponding author on reasonable request.
